# An image guided small animal stereotactic radiotherapy system

**DOI:** 10.18632/oncotarget.7939

**Published:** 2016-03-06

**Authors:** Hao Sha, Thirupandiyur S. Udayakumar, Perry B. Johnson, Nesrin Dogan, Alan Pollack, Yidong Yang

**Affiliations:** ^1^ Department of Radiation Oncology, University of Miami School of Medicine, Miami, FL, 33136, USA

**Keywords:** computed tomography (CT), contrast enhancement, image guidance, stereotactic radiotherapy, preclinical radiation research

## Abstract

Small animal radiotherapy studies should be performed preferably on irradiators capable of focal tumor irradiation and healthy tissue sparing. In this study, an image guided small animal arc radiation treatment system (iSMAART) was developed which can achieve highly precise radiation targeting through the utilization of onboard cone beam computed tomography (CBCT) guidance. The iSMAART employs a unique imaging and radiation geometry where animals are positioned upright. It consists of a stationary x-ray tube, a stationary flat panel detector, and a rotatable and translational animal stage. System performance was evaluated in regards to imaging, image guidance, animal positioning, and radiation targeting using phantoms and tumor bearing animals. The onboard CBCT achieved good signal, contrast, and sub-millimeter spatial resolution. The iodine contrast CBCT accurately delineated orthotopic prostate tumors. Animal positioning was evaluated with ∼0.3 mm vertical displacement along superior-inferior direction. The overall targeting precision was within 0.4 mm. Stereotactic radiation beams conformal to tumor targets can be precisely delivered from multiple angles surrounding the animal. The iSMAART allows radiobiology labs to utilize an image guided precision radiation technique that can focally irradiate tumors while sparing healthy tissues at an affordable cost.

## INTRODUCTION

In radiotherapy, clinicians and researchers rely on translational studies performed on laboratory animals to investigate radiobiology hypothesis, test new biomarkers and develop novel therapeutics. Preferably, these studies should be performed on irradiators capable of delivering focal irradiation with image guidance [1, 2]. Thus far, two products have been commercialized for this purpose, including the SARRP system developed at Johns Hopkins University [3] and the X-Rad 225Cx system developed at Princess Margaret Hospital [4]. Both systems utilize cone beam CT (CBCT) for target localization, and their introduction has already led to exciting findings in radiobiology studies [5, 6]. The CBCT on both commercial systems relies upon the traditional design of a rotating gantry which houses both an x-ray source and/or detector [1, 3, 4, 7]. The complexity of this design warrants nontrivial efforts for periodic calibration and maintenance. The cost of these systems can also be a prohibitive factor hindering their wide scale adoption.

A new type of small animal irradiator, an image guided SMall Animal Arc Radiation Treatment system (iSMAART), has been developed. It employs a unique imaging and radiation geometry where animals are positioned upright, and holds the x-ray source and flat panel detector stationary while rotating the animal in the vertical position. This design has several advantages. Of note, the removal of a rotating gantry reduces manufacturing cost, improves stability, and simplifies operation. Furthermore, the radiation beam projects in only a single direction which simplifies radiation shielding and reduces the total weight of the device. The system was built on a mobile platform which could be conveniently adopted by radiobiology researchers in a limited lab space. The calibration and validation of the iSMAART system is presented in this work. Specific tests were designed and experiments carried out to evaluate its imaging and treatment performance using phantoms and tumor bearing animals. Of particular interest was the stability of the specimen during treatment as the upright animal positioning is not commonly used and poses unique challenges.

## RESULTS

### Imaging

After the CBCT geometrical calibration, the source-to-axis distance (SAD) and source-to-detector distance (SDD) were calculated to be 342.6 mm and 536.4 mm, respectively. This led to an image magnification of 1.57 and an achievable maximum field of view of 13 × 13 × 13 cm^3^. The projection of the central axis onto the imaging panel (u_0_, v_0_) was located at the 536.4 pixel along the horizontal direction and the 464.6 pixel along the vertical direction. The in-plane rotation of the imaging panel was found to be 0.18 degree in the counterclockwise direction.

**Figure 1:**
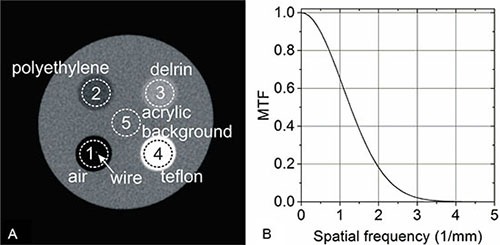
CBCT Image quality (**A**) A transverse plane of the CBCT image of the Image quality phantom. There are five heterogeneities imbedded inside the acrylic cylinder. Regions of interest (ROIs) were placed on each heterogeneity for signal to noise ratio (SNR) and contrast to noise ratio (CNR) analysis: air, polyethylene, delrin, teflon, and acrylic background (from 1 to 5). The arrow points to the 50 μm radiopaque wire stretched through the air heterogeneity for spatial resolution measurement. (**B**) The modulation transfer function (MTF) derived from a wire based oversampling method.

Figure 1A shows an axial CBCT slice of the acrylic phantom used during image quality testing. The measured signal-to-noise ratios and contrast-to-noise ratios are shown in Table 1. The SNRs for polyethylene, delrin, teflon and acrylic background were 21.8 ± 0.7, 32.4 ± 1.1, 48.3 ± 1.9 and 25.8 ± 1.3, respectively. The CNRs for air, polyethylene, delrin and teflon were −24.2 ± 1.2, −5.5 ± 0.3, 5.7 ± 0.3, and 24.4 ± 1.2, respectively.

**Table 1:** SNRs and CNRs for CBCT

ROI	SNR	CNR
Air	NA	− 24.2 ± 1.2
Polyethylene	21.8 ± 0.7	− 5.5 ± 0.3
Delrin	32.4 ± 1.1	5.7 ± 0.3
Teflon	48.3 ± 1.9	24.4 ± 1.2
Acrylic background	25.8 ± 1.3	NA

Also shown in Figure 1A is the wire stretched through the air cylinder which was used to determine the modulation transfer function (MTF). The full width at half maximum (FWHM) obtained from the Gaussian curve fitting (*R*^2^ = 0.92) of the oversampled line profile across the wire was 0.35 mm. Since the physical diameter of the wire (50 μm) was much smaller than the measured FWHM value, the wire was deemed thin enough for the spatial resolution measurement. The measured MTF is shown in Figure 1B. The spatial frequencies were f_0.1_ = 2.3 mm^−1^ at 10% amplitude and f_0.02_ = 3.0 mm^−1^ at 2% amplitude.

### Dosimetry

**Figure 2:**
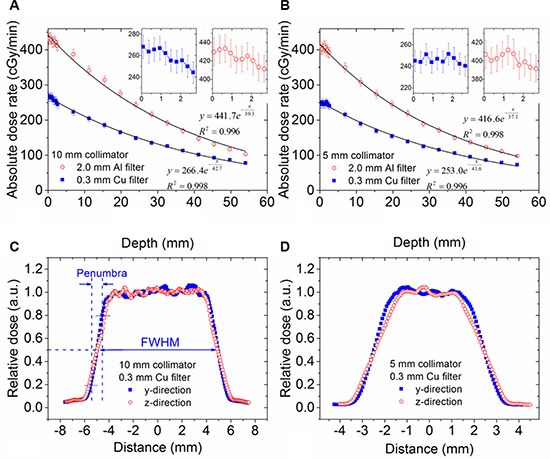
The depth dose and cross-sectional dose profiles The depth dose profiles for 10 mm collimator (**A**) and 5 mm (**B**) respectively. X-ray was filtered with 2.0 mm Al or 0.3 mm Cu. Two inserts in each panel show the entrance dose measurements from 0.15 to 2.85 mm with stacked EBT films. (**C** and **D**) show the cross-sectional dose profiles measured at 11.2 mm depth with 0.3 mm Cu filter for 10 and 5 mm collimator respectively. The definition of penumbra is illustrated in (c).

The absolute depth dose measured with EBT2 film is shown in Figure 2A and 2B. The entrance dose rate was measured from the ten films stacked at the phantom surface (displayed as inserts in Figure 2A and 2B), and the remaining depth dose points from the films sandwiched inside the phantom. The absolute dose rate was found to be an exponential function of measurement depth (*R*^2^ = 1.00 for all curve fittings). No obvious skin sparing was observed at the entrance of the 225 kVp x-ray beams.

Two cross section profiles are shown in Figure 2C and 2D. Both profiles correspond to the isocenter plane at a depth of 31.5 mm. Variations in the plateau region are due to noise in the film measurement. The FWHMs and penumbras for the radiation beams are shown in Table 2. The beam diameters at the isocenter plane represented by FWHM values were 10.9 mm for the 10 mm collimator and 5.5 mm for the 5 mm collimator. The standard deviation of the FWHM measurement was negligible. The beam penumbra was 1.1 mm along the y direction and 1.5 mm along the z direction for both the 10 mm and 5 mm collimators.

**Table 2:** The FWHM and penumbra for 10 mm and 5 mm collimators

	10 mm collimator		5 mm collimator	
	y direction	z direction	y direction	z direction
FWHM (mm)	10.9	10.9	5.5	5.5
Penumbra (mm)	1.1	1.5	1.1	1.5

Note: Measurements were repeated three times. The standard deviation was negligible.

**Figure 3:**
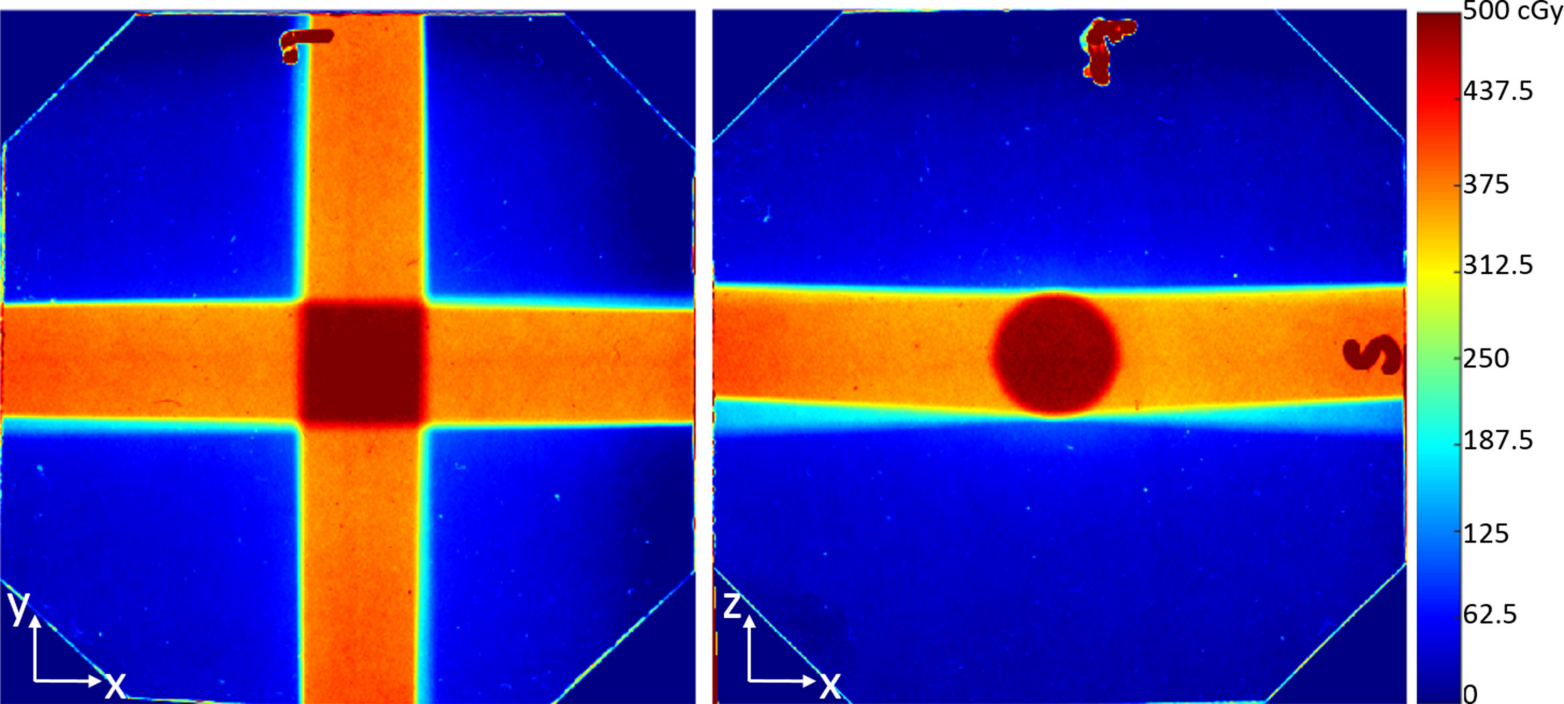
The dose distribution from 4 evenly separated beams A total of 5 Gy dose was delivered to the center of a water equivalent cubic phantom of 63 × 63 × 63 mm^3^ size. (Left and right images) are the dose distribution in the x-y plane, and x-z plane, respectively. Dose distribution was obtained from EBT2 films inserted in the phantom.

Figure 3 shows the dose distribution from the conformal dose delivery of 4 beams to the cubic phantom. As expected, a four field box pattern was seen in the x-y plane, and a round area pattern was seen in the x-z plane. The size of the high dose area (5 Gy) in the x-y plane was 11.0 × 11.0 mm^2^, and the diameter of the high dose area in the x-z plane was 11.1 mm (consistent from both x and z direction). In this case, the 5 Gy isodose line would sufficiently cover a tumor whose longest length is close to but smaller than 11.0 mm, excluding uncertainties from animal positioning and mechanical movements.

### Image guidance

**Figure 4:**
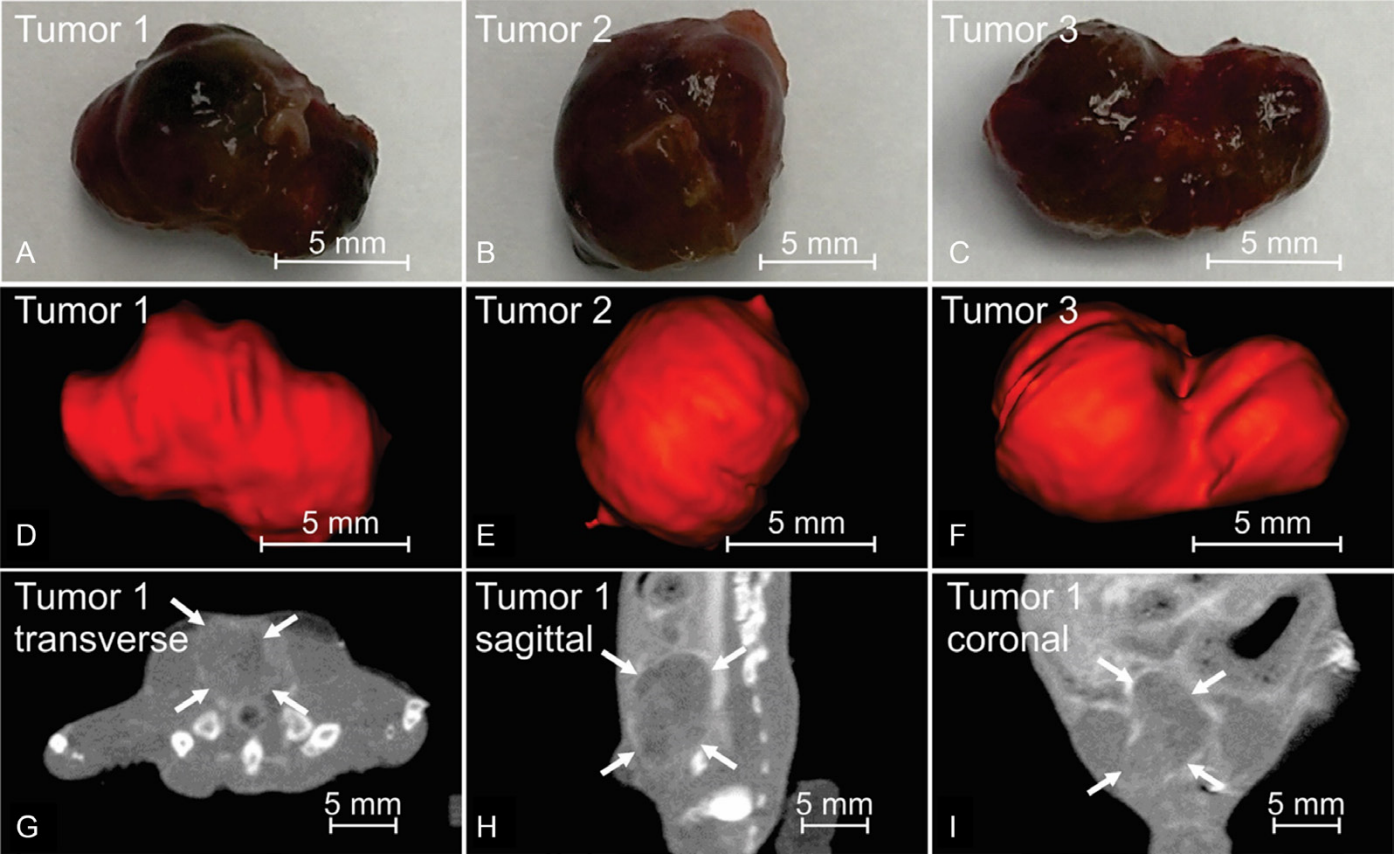
Contrast enhanced CBCT of orthotopically grown LNCaP prostate tumors Panel (**A–C**) show the LNCaP prostate tumors excised immediately after the animal was euthanized, with dark red color indicating rich vasculature. (**D–F**) are the three-dimensional rendering of corresponding tumors contoured from CBCT scanned immediately before animal sacrifice. (**G–I**) are the three orthogonal CBCT slices of tumor 1, with arrows pointing to the enhanced tumor edges. Comparison between (a)-(c) and (d)-(f) proves a good match between the CBCT contours and actual tumor volumes.

LNCaP Prostate tumors grew in four out of five mice, which was validated after tumor excision. The prostate tumor in one mouse was too small to correctly identify in the contrast CBCT. In this case the volume was 12.6 mm^3^ with a length of less than 3 mm along the longest side. For the remaining three mice, tumors were identified in the contrast CBCT and compared to the caliper based measurements (Table 3). The difference between the two measurement methods ranged from 3.4 to 4.4%. The photographs of the three tumors after excision are shown in Figure 4A–4C. The dark red color indicates rich vasculature inside these LNCaP prostate tumors. The 3D tumor renderings from CBCT are shown in Figure 4D–4F. As seen in the figure their shapes match closely those of the excised tumors. As an example, the orthogonal planes from the contrast CBCT of one prostate tumor are shown Figure 4G–4I. We can clearly visualize the tumor edge that was more enhanced than its internal area. The arrows in Figure 4G–4I point to the enhanced tumor edge.

**Table 3:** Tumors volumes from CBCT and caliper measurement

	Tumor 1	Tumor 2	Tumor 3	Tumor 4
CBCT (mm^3^)	346.2	498.9	434.7	NA
Caliper (mm^3^)	331.6	478.2	449.8	12.6
Difference (%)	4.4	4.3	3.4	NA

Note: Tumor 4 was too small to be recognized with contrast CBCT.

### Targeting performance

#### Movement precision of animal stage

**Figure 5:**
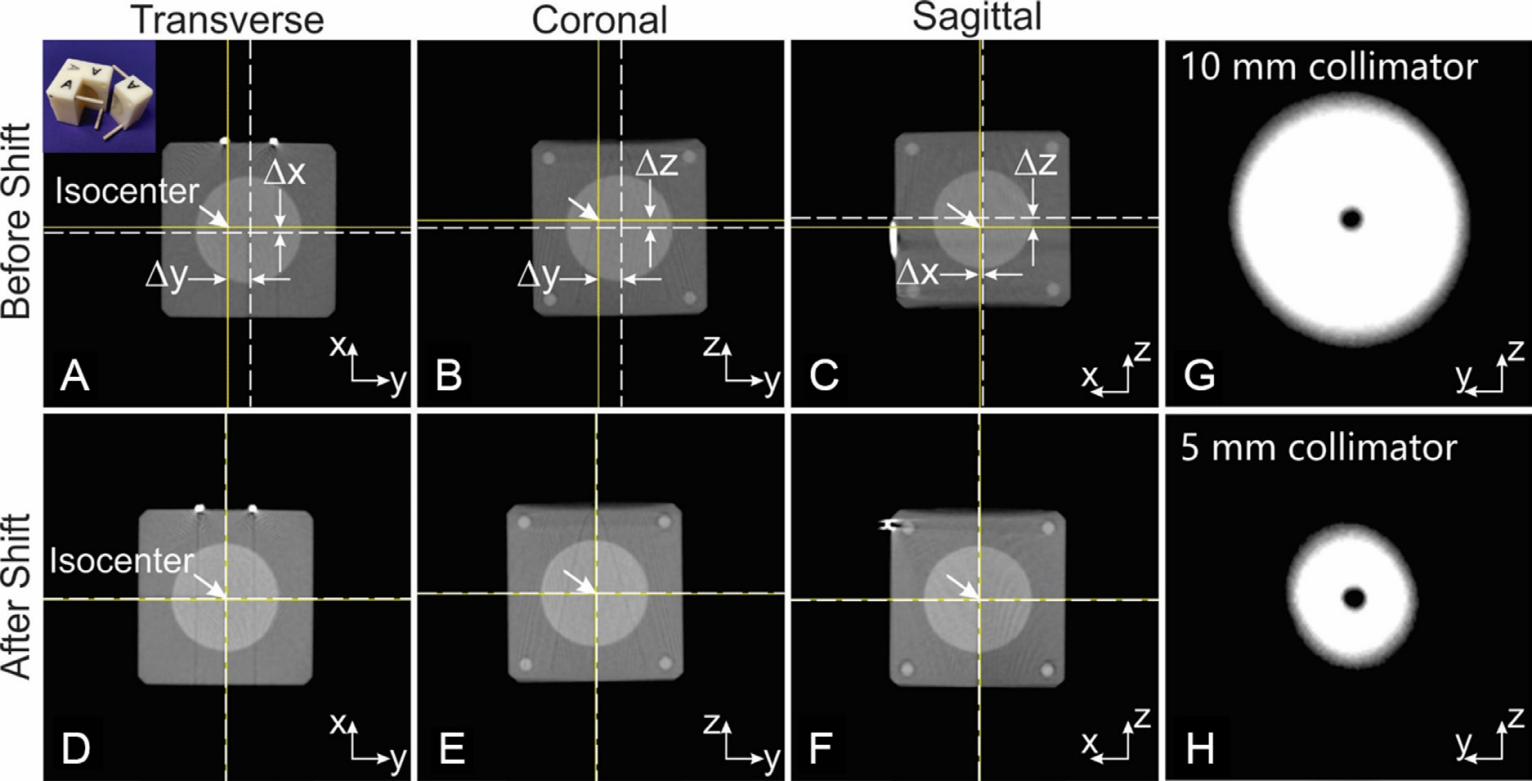
The mechanical precision test for the animal stage translation and collimator positioning (**A–C**): the orthogonal CBCT views of the mini-ball cube phantom before shift. The solid crosshair indicates the imaging/radiation isocenter, and dash crosshair the ball center. Δx, Δy, Δz are 0.39, 1.56 and 0.52 mm respectively. The ball center was aligned to the isocenter with only one stage adjustment. (**D–F**) confirmed the coincidence of isocenter and ball center after shift. The insert in the first panel is a photo of the mini-ball cube phantom. (**G** and **H**) are the x-ray exposures of a radiopaque ball centered at isocenter through 5 and 10 mm collimator respectively. The ball center and center of x-ray field are well aligned, indicating the coincidence of the isocenter and central axis of collimated radiation beams. The imperfect round shape of the collimated beam may be caused by some residual lead left on the inner edge of the collimator aperture.

The offsets of the mini-ball cube phantom relative to the isocenter before and after a single translational adjustment (one shift in each direction) are demonstrated in Figure 5A–5F with orthogonal CBCT views. The bright spots in the axial and sagittal views are from a V-shaped steel wire attached to the phantom surface for identification of the laboratory coordinates. The isocenter is indicated by the crosshair of the solid lines, and the ball center is indicated by the crosshair of the dashed lines. The offsets between the ball center and the isocenter before shift were 0.39 mm, 1.56 mm and 0.52 mm along the x, y, and z directions, respectively. After shifting, the residual offset was < 0.13 mm (one voxel size in CBCT) as measured from CBCT. The test procedure was repeated three times by three different lab members, with initial offsets intentionally >10 mm along each direction. In each case, the residual offset after shift adjustment were consistently < 0.13 mm.

### Alignment precision of collimator

In the collimator alignment test, the averaged deviations between the center of the ball and the center of the collimated exposure for all collimators were 0.17 ± 0.09 mm along the horizontal direction (y direction) and 0.00 ± 0.04 mm along the vertical direction (z direction). This resulted in a radial distance of 0.17 ± 0.09 mm. Projection images from the alignment test of the 5 mm and 10 mm collimators are shown in Figure 5G and 5H. In this example, the radial deviation between the isocenter and the beam central axis was 0.13 mm for the 10 mm collimator and 0.26 mm for the 5 mm collimator.

### Positioning precision of animal setup

**Figure 6:**
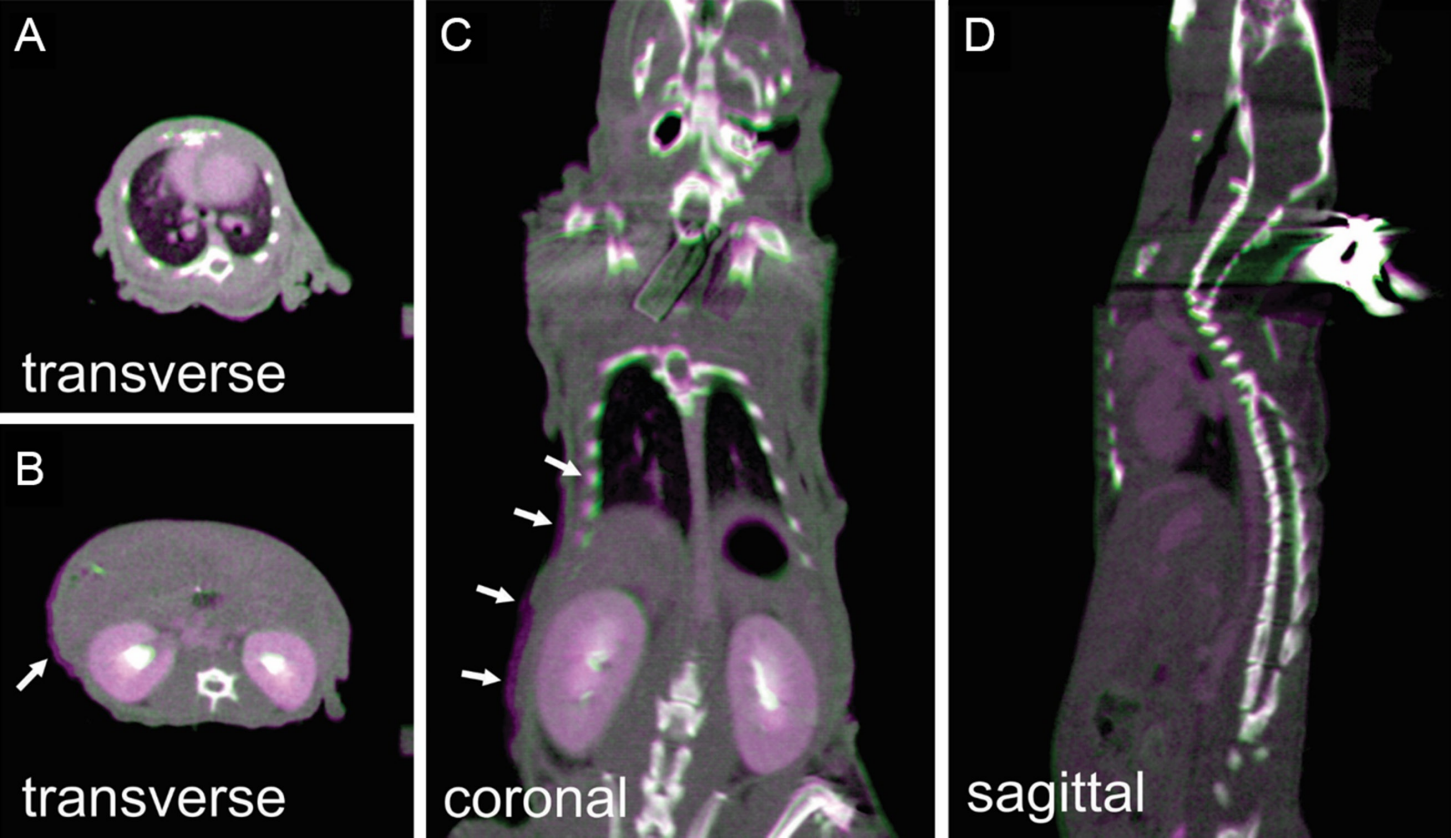
The pseudocolor overlay of mouse CBCTs acquired immediately (purple) and 15 minutes (green) after animal setup on the stage The animal received iodine contrast injection before imaging. (**A–D**) are one transverse slice at thorax region, transverse slice at abdominal region, coronal slice, and sagittal slice, respectively. Arrow in (**B** and **C**) point to the slight mismatch at the ribs and abdominal skin contours.

The test results for the positioning of the anesthetized mice are summarized in Table 4. The position variances during a period of 15 minutes were < 0.5 mm for all anatomical regions. The mean variances were 0.21 ± 0.16 mm, 0.20 ± 0.21 mm, and 0.31 ± 0.27 mm along the anterior-to-posterior (AP), left-to-right (LR) and superior-to-inferior (SI) directions, respectively. The maximum variance was approximately 0.43 mm which occurred along the SI direction at the thorax region. Figure 6 shows the image overlay of two CBCTs acquired at the beginning of the procedure and 15 minutes after a mouse had been immobilized on the animal stage. The match between the two image sets indicates that the animal positioning during the 15 minute period was reasonably stable. There was noticeable misalignment particularly in the lung and abdominal regions indicated by the non-perfect match for the ribs and the abdominal skin contours. As these two regions are particularly susceptible to respiratory motion, it is likely that temporal averaging of the CBCT over the respiratory phase played some role in this small misalignment. Other major organs such as the brain, heart, liver, and kidney were all aligned reasonably well.

**Table 4:** Variances of animal position during a period of 15 minutes

Direction	Thorax (mm)	Abdomen (mm)	Pelvis (mm)	Mean (mm)
AP	0.21 ± 0.18	0.20 ± 0.15	0.23 ± 0.17	0.21 ± 0.16
LR	0.15 ± 0.14	0.27 ± 0.27	0.18 ± 0.16	0.20 ± 0.21
SI	0.43 ± 0.31	0.18 ± 0.15	NA	0.31 ± 0.27

Note: AP, LR, SI are anterior-to-posterior, left-to-right, and superior-to-inferior directions, respectively. Data was not acquired in the pelvis region along SI direction.

## DISCUSSION

In the present study, a bench top small animal irradiator, iSMAART, has been developed. It provides a solution for CT guided precision stereotactic radiation at affordable cost by employing a unique imaging and radiation geometry. The design avoids the rotating gantry for CT imaging or stereotactic radiation delivery as used in other systems [1, 3, 4, 7], and consequently simplifies mechanical control and improves stability. The light weight of the animal on the stage allows the animal to be rotated fast for accelerated CBCT acquisition, which is important in pharmacokinetics studies [8] and may not be feasible in the rotating gantry configuration.

The design requires a special animal setup where the animal is positioned *upright* instead of prone as often seen on other systems. On one hand, the upright geometry actually mimics the conventional micro-CT geometry where the x-ray gantry and detector panel rotate around the long axis of the prone positioned animal, and thus preserves the image quality [9]. On the other hand, the upright geometry allows the shortest beam paths through normal tissues from multiple angles around the animal and thus ensures the effective delivery of radiation beams to the tumor target while maximally sparing normal tissues. Although the upright positioning is uncommon, researchers have used it in imaging [10, 11] and irradiation of rodent animals [12]. The positioning test demonstrated acceptable animal stability during the entire image guided radiation procedure. Nonetheless, one should bear in mind that this test was performed with the animals being taped on a flat plate which represents the worst setup scenario, and that the positioning stability can be further improved using a body-fitted holder [13, 14].

Besides the upright animal positioning, several other issues are worth discussion. First, CT is necessary to provide image guidance for the precise delivery of radiation beams [2], but, it is not able to differentiate tumor from its surrounding soft tissues without contrast agent injection [15]. The ring-enhancement pattern in the iodine contrast CT, which has been reported for various tumor types [16–18], enabled us to differentiate and delineate the LNCaP prostate tumor from its surrounding tissues and to directly target the tumor with radiation beams. However, the iodine contrast may not work for other tumor types, particularly those poorly vascularized. Second, the depth dose curve fitting approximated to a simple exponential attenuation, which can simplify dose calculation. A practical treatment planning system accounting for tissue heterogeneity in dose computation is under development. Third, we commissioned two x-ray filters. The 0.3 mm Cu filter produced a harder x-ray beam and consequently slower dose fall-off with depth. Therefore the Cu filter can be selected for irradiation of deep seated tumors in large animals such as rat and rabbit while the 2 mm Al filter is appropriate for mice irradiation.

The iSMAART achieved an overall targeting uncertainty of < 0.38 mm, consisting of < 0.13 mm in animal stage movement, 0.17 mm in collimator alignment, and 0.31 mm in animal positioning. A 0.5 mm margin expansion from the tumor volume would sufficiently account for this uncertainty and ensure full target coverage. With the current version, an entire image guided irradiation process takes about 10 minutes: ∼2 minutes for CBCT; ∼5 minutes for animal stage adjustment; and ∼3 minutes for irradiation of a typical tumor with 5 Gy dose. The efficiency will be further improved after we motorize the animal stage and seamlessly integrate the experimental workflow.

## MATERIALS AND METHODS

### System configuration

**Figure 7:**
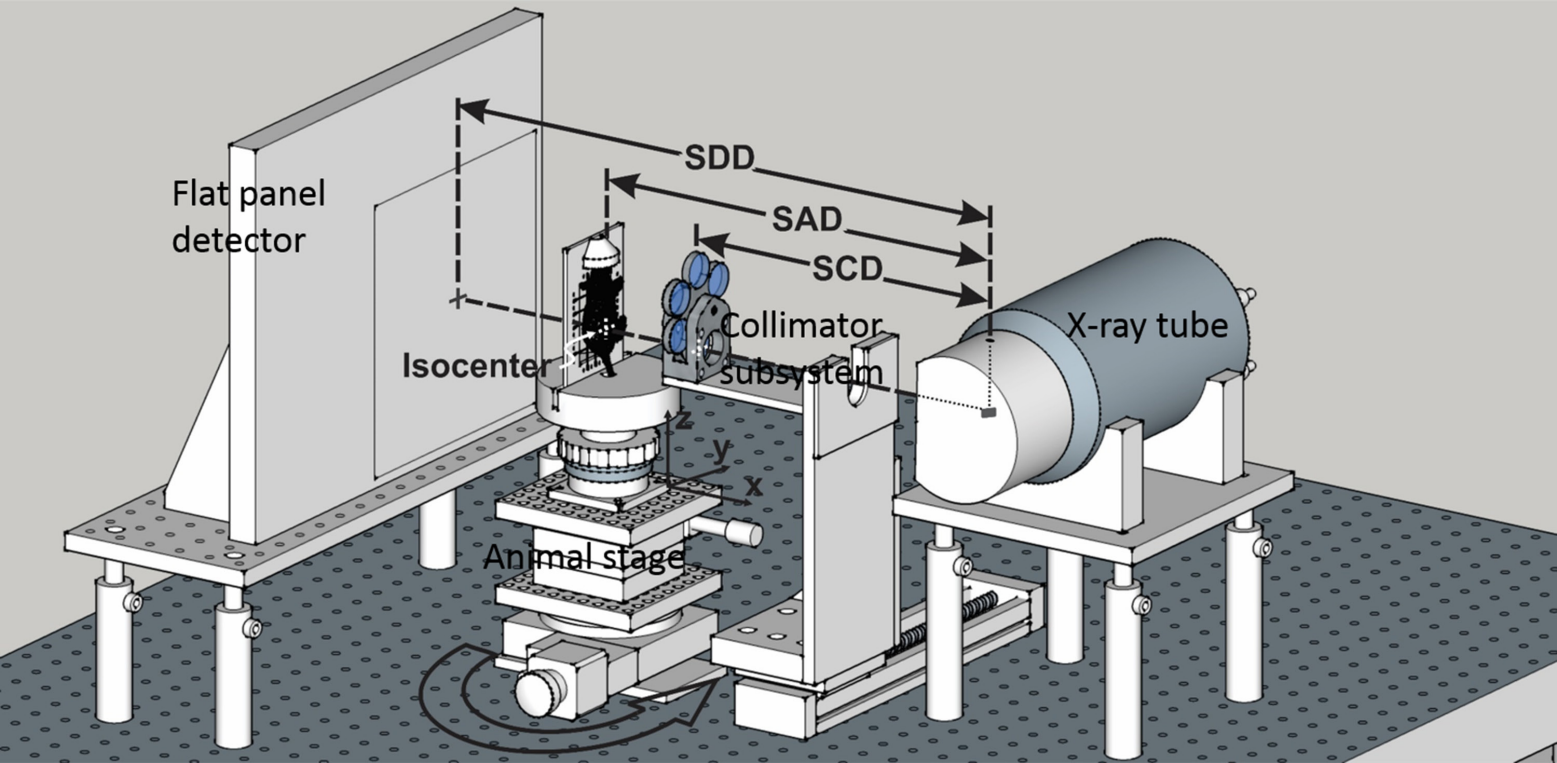
The iSMAART configuration The x-ray tube and flat-panel detector are fixed on the optical bench and the animal stage can be translated along x-y-z directions and rotated around z-axis. The anesthetized mouse is positioned upright with a customized animal holder. The motorized collimation subsystem is positioned in or out of the radiation for treatment or imaging. The imaging and irradiation isocenters are coincident with each other. The nominal values for the source to detector distance (SDD) is 52.5 cm, source to axis distance (SAD) 35 cm, and source to collimator distance (SCD) 25 cm.

The iSMAART research platform consists of a stationary x-ray source and flat-panel detector, a movable animal stage with four degrees of freedom (x-y-z translations and z axis rotation; x axis is defined as the direction from the imaging panel to the x-ray tube along the beam central axis, z axis as the vertical direction towards the ceiling, and y axis as the direction perpendicular to both the x and z axis) and a customized collimation subsystem (Figure 7). The imaging/treatment isocenter is defined as the intersection of the rotating vertical axis and the central axis of the radiation beam. The x-ray source (COMET AG, Flamatt, Switzerland) has a nominal tube voltage of 225 kV and dual focal spots of 1.0 mm and 5.5 mm (EN12543 standard) corresponding to continuous ratings of 640 W and 3000 W, respectively. The small focal spot is used for CBCT imaging and large spot for radiation treatment. The 20 cm × 20 cm a-Si detector (PerkinElmer, Waltham, MA, USA) has a 200 μm pixel resolution, and can sustain a frame rate of 25 Hz at full resolution (up to 100 Hz after binning). This ultrafast image acquisition provides 4D-CBCT capability which is especially important when studying small animal respiratory motion. The animal stage (Thorlabs, Newton, NJ, USA) is capable of 360° continuous rotation at a speed up to 50° per second with 0.08° accuracy.

The collimation subsystem is driven by a motorized linear actuator (NEMA 17 DC stepper motor and Motor-Mount Slides, McMaster-Carr, Elmhurst, IL, USA) which can be activated remotely and has a precision of 0.0002 inches per inch of travel. During imaging, the entire subsystem is positioned aside to clear the radiation path. This configuration is called the “imaging position”. The “treatment position” is then set by re-inserting the collimation subsystem into the radiation path and selecting an appropriately sized collimator. For this selection there are six lead collimators available which define circular apertures of 3, 5, 7, 8, 10, and 15 mm diameters. Each collimator is located on a rotatable carousel which pivots around a horizontal axis. A spring-loaded detent mechanism in the collimator wheel locks the collimators with the central axis of the collimated beam aligned to the imaging/treatment isocenter. The nominal value for the source-to-collimator distance (SCD) is 25 cm. The system control, such as x-ray on/off, image acquisition, and stage rotation, is performed on a computer with a 64-bit operating system, an Intel Core i7-4770 3.4 GHz CPU, 32 GB RAM, and NVIDIA Quadro K2000 2 GB GPU. The parameters for CBCT imaging and radiation treatment are presented in Table 5.

**Table 5:** Imaging and treatment parameters on iSMAART

Parameter	Imaging	Treatment
Tube voltage (kVp)	45/65	225
Tube current (mA)	5.5/1.2	13
Focal spot (mm)	1.0	5.5
Filtration	0.1 mm Cu	0.3 mm Cu or 2 mm Al

### Imaging

The CBCT subsystem on board the iSMAART incorporates the x-ray tube, rotation animal stage, and flat panel detector. The subject to be imaged is positioned upright on the rotation stage and rotated around the vertical axis between the stationary x-ray source and the detector panel. The nominal values for the SDD and SAD are 52.5 cm and 35.0 cm, respectively, resulting in a nominal image magnification of 1.5 at the detector panel. The imaging beam is produced using the small x-ray focal spot of 1.0 mm, rated at 45 kVp and 5.5 mA, or 65 kVp and 1.2 mA. A copper filter (0.1 mm thickness) is used to remove low energy photons and reduce the imaging dose to the animal. CBCT scans are acquired with 360 projections during one full rotation (one projection per 1° rotation). Several corrections are automatically incorporated during image acquisition including corrections for offset, gain, and bad pixels. Images are reconstructed with 0.13 mm × 0.13 mm × 0.13 mm voxels using the FDK filtered back projection reconstruction algorithm [19].

The imaging sub-system was geometrically calibrated with a method initially proposed by Noo *et al* [20, 21]. A calibration phantom incorporating seven steel beads was scanned using 36 projections (one projection per 10° rotation). The diameter of each bead was 3.2 mm and all beads were evenly distributed along a vertical acrylic rod. The projection data was analyzed with an in-house program written in MATLAB (MathWorks, Natick, MA, USA). The following parameters were calculated: SDD and SAD; projection of the x-ray beam central axis on the detector panel, u_0_ and v_0_, where u_0_ is the distance along the horizontal axis and v_0_ the distance along the vertical axis of the panel; detector panel in plane rotation angle, θ, where the rotation is defined in a plane perpendicular to the x-ray beam central axis; field of view and image magnification.

Image quality was assessed using a custom made phantom. The phantom was fabricated from an acrylic (1.19 g/cm^3^) cylinder 75 mm in length and 32 mm in diameter. Four cylindrical inserts were imbedded inside the acrylic as shown in Figure 1A. Each insert was 6 mm in diameter and composed of a different material including air (0.0012 g/cm^3^), polyethylene (0.94 g/cm^3^), delrin (1.41 g/cm^3^), and teflon (2.21 g/cm^3^). The phantom was positioned upright for image acquisition with its long axis parallel to the vertical axis of the system. Three CBCTs were collected using a potential of 65 kVp and a current of 1.2 mA.

In order to quantify the scans using common image quality metrics, the open source software ImageJ (National Institutes of Health, Bethesda, MD, USA) was used to draw a circular region of interest (ROI) within each insert. Each ROI had a diameter of roughly 40 pixels and was drawn on an axial slice of the CBCT. The SNR, CNR and spatial resolution were calculated. The SNR was defined as SI_ROI_ /σ_ROI_, where SI_ROI_ was the mean signal intensity of an ROI, and σ_ROI_ the corresponding standard deviation. The CNR was defined as (SI_ROI_ – SI_bkg_)/σ_bkg_, where SI_bkg_ was the mean signal intensity of one same size ROI drawn in the background, and σ_bkg_ was the standard deviation of the background. The results are presented in the format of mean ± standard deviation.

The spatial resolution was estimated with a wire based oversampling method which has been used previously for the measurement of CBCT spatial resolution [22, 23]. For the current study, a 50 μm diameter stainless steel wire was stretched through the air insert found inside the acrylic phantom. The wire was tilted slightly by approximately five degrees relative to the long axis of the phantom for oversampling purposes. Figure 1A shows an axial CBCT slice of the phantom with the wire displayed as a bright spot within the air ROI. ImageJ was used to measure horizontal profiles across the wire which were sampled over 20 adjacent axial slices. A Gaussian function was used to fit the average profile and obtain the line spread function. The MTF was then derived via Fourier transform of the line spread function.

### Dosimetry

The therapy beam of the iSMAART system is produced using the large x-ray focal spot of 5.5 mm, rated at 225 kVp and 13 mA. Either a 0.3 mm thick Cu or a 2 mm thick Al filter is employed to harden the x-ray beam and improve its penetration. In order to quantify common dosimetric parameters for this beam, several measurements were made using Gafchromic EBT2 QD+ films (Ashland Inc., Covington, KY, USA). The EBT2 films were calibrated using a ^60^Co radiation source from a clinical Gamma Knife radiotherapy unit (Perfexion, Elekta, Stockholm, Sweden). Briefly, a series of 5 × 5 cm^2^ EBT2 films were irradiated on the Gamma Knife at 13 different dose levels ranging from 0.5 to 12.0 Gy. Films were scanned as color images two days after exposure in an Epson Perfection V700 photo scanner (Long Beach, CA, USA) with an unexposed film scanned as the baseline. All images were analyzed in ImageJ where the red channel of each color image was extracted for analysis. Each film was scanned three times and the average of the three readings was calibrated against the known radiation dose. The calibration curve was fitted using a polynomial function and used to calculate radiation dose as measured by EBT2 films irradiated using the iSMAART system.

Depth dose and cross-sectional dose profiles were measured in a film stack cubic phantom (CIRS, Norfolk, VA, USA) which was 63 × 63 × 63 mm^3^ in dimension and composed of PWDT water equivalent material [24]. The films were stacked inside the phantom at increments of 4.3 mm (4 mm phantom material plus 0.3 mm film thickness). To measure the x-ray entrance dose with a high depth resolution, another 10 films were stacked together and attached to the front surface of the phantom, resulting in a total of 23 films in each measurement. This configuration provided entrance dose measurements at depths of 0.15 mm to 2.85 mm using 0.3 mm increments (the measurement locations were defined at the middle of each film thickness). The entire phantom may be considered water equivalent as both film [25] and phantom [24] are water equivalent at the x-ray energy used in this study. Two collimators were tested with aperture sizes of 5 mm and 10 mm and using two different filtering settings of 0.3 mm Cu and 2 mm Al, respectively. All films were processed consistently with the calibration procedures described previously. The x-ray beam penumbra derived from the cross-sectional dose profiles was defined as the distance between two points located at 20% and 80% of the maximum intensity on the same side.

To confirm the iSMAART's capability of conformal dose delivery with radiation beams from multiple angles, a dose of 5 Gy was delivered to the center of the cube phantom (31.5 mm depth) with 4 beams (1.25 Gy from each beam) by rotating the stage 0°, 90°, 180° and 270° respectively. CBCT guidance was used to align the center of the phantom to the imaging/treatment isocenter before irradiation. EBT2 films were inserted at the center plane of the phantom to measure the dose distribution in the x-y plane and x-z plane. The radiation delivery was repeated twice. During the first irradiation, the cubic phantom was positioned such that the film was oriented in the x-y plane; during the second irradiation, it was positioned such that the film was oriented in the x-z plane. In both situations, the cubic phantom was such aligned that either the x, y or z axis was normal to each of its surface, therefore the dose distribution in the y-z plane would be similar to that in the x-z plane.

### Image guidance

In order to test the image guidance ability for small animal subjects, five live mice were imaged (12 weeks old, 20–25 g body weight). Each mouse had previously been inoculated with LNCaP prostate tumor cells in the prostate as described in detail earlier [26] and was anesthetized during imaging through intraperitoneal injection of a ketamine: xylazine cocktail (100:10 mg/kg body weight). The animal was immobilized on a specially designed animal holder which was anchored to the animal stage. The holder was comprised of a hollow, rectangular acrylic plate with nylon wires affixed crossly for body support and a nose cone attached on the top edge. All experiments were performed with the approval of the University Animal Care and Use Committee.

Of particular interest during this study was the potential for applying an iodine based contrast agent to enhance tumor visualization and improve image guidance. To this end, each mouse received an intravenous injection of Iopamidol at 1000 mg/kg of body weight via tail veins. Iopamidol is an FDA approved iodine based CT contrast (Bracco Diagnostics, Princeton, NJ, USA). CBCT images were acquired before and immediately after contrast injection. After contrast injection, each animal was continuously monitored with repeat CBCT scans for up to 30 minutes. To further enhance the contrast caused by the iodine, the lower energy x-ray beam was selected for imaging with settings of 45 kVp, 5.5 mA, and 0.1 mm Cu filter.

To validate tumor visualization by CBCT, each mouse tumor was contoured using ImageJ and compared by shape and volume with those excised from the animals. Each mouse was immediately sacrificed after the final CBCT. The dimension of each excised tumor was measured with a caliper along its length (L), width (W), and height (H) with L corresponding to the longest dimension. The tumor volume (V) was estimated with the formula V = (1/6) π (L) (W) (H) assuming an ellipsoid approximation. In CBCT, the tumor contours were drawn along the enhanced tumor edges in the axial plane and displayed with 3D rendering. The volumes measured from the final CBCT were compared with those measured with a caliper after tumor excision.

### Targeting performance

The uncertainty of radiation targeting for the iSMAART system is determined by three major components: the movement precision of the animal stage, the alignment precision of the collimation subsystem, and the positioning precision of the animal setup. In order to quantify the first of these three components, a mini-ball cube phantom (Accuray Inc., Sunnyvale, CA, USA) was imaged after being aligned with an obvious offset from isocenter. The phantom, comprised of a 3 cm plastic cube imbedded with a 1.8 cm diameter acrylic sphere, was then shifted to correct the setup error as identified by the CBCT image guidance system. A post-shift CBCT was acquired to confirm the accuracy of the movement.

Next, the alignment precision of the collimation subsystem was assessed by targeting a radiopaque steel ball immobilized on an acrylic plate and positioned at isocenter using CBCT image guidance. The collimation subsystem was driven from the imaging position to the treatment position. The collimator wheel then was rotated at least one full rotation before a chosen collimator was fixed in the radiation beam path. An x-ray projection image was acquired using the therapeutic beam and the flat panel detector. The entire procedure was repeated until all six collimators were tested. The distance between the center of the steel ball and center of the collimated exposure was obtained from the projection images and back-projected to the isocenter plane.

Finally to assess the positioning precision of the animal setup, a mouse was sedated and immobilized on the animal holder and continuously monitored with CBCT for 15 minutes, during which animals were rotated for CBCT acquisition. The difference in animal positioning between the first and last CBCT scan was calculated using landmark analysis. Landmarks were chosen in three body regions (thorax, abdomen, and pelvis) along three orthogonal directions (AP, LR and SI directions). In each region, two perpendicular line profiles were drawn on each of three orthogonal planes, across the heart and thoracic spine in the thorax, kidney and lumbar spine in the abdomen, and tumor in the pelvis. Peaks and valleys in each line profile were identified and their centers used to calculate the anatomical shifts between the first and last time points. To visually check the shift, if any, the first and last CBCT scans were overlaid and displayed with a pseudocolor blending. The first scan was displayed in purple and the last scan was displayed in green. This experiment was repeated for seven different mice with all image processing being done in MATLAB.
